# Panel Practitioners and Chemists

**Published:** 1914-07-25

**Authors:** 


### Panel Practitioners and Chemists.

### EXCESSIVE CALLS UPON THE DRUG FUND.

The relations of panel practitioners with chemists principally occupied the newly-elected Panel Committee for London at its meeting on July 21. Dr. H. J. Cardale was elected chairman of the committee for the year, Dr. J. A. Angus, vice-chairman, and Dr. Lauriston E. Shaw, treasurer.

The Committee gave informal consideration to an extensive list, which came before the London Insurance Committee on Thursday, of preparations which it is proposed shall not be prescribed at the cost of the Drug Fund. The view was expressed that, although the meeting was in sympathy with the prohibition of practically all the items on the list, the Panel Committee should have been consulted before the Insurance Committee was recommended to take definite action. The Panel Committee representatives on the Insurance Committee were asked to bring this opinion before that body.

With only four dissentients, the Committee agreed to limit the right of practitioners to prescribe in the form "Rep. mist.," the chemists having made strong representations on the subject. The meeting agreed that until an official Pharmacopœia is recognised by the bodies working the Act, or some modification is made in the method of ordering drugs and appliances, practitioners should be permitted to use "Rep. mist.," provided that the date of the original prescription is given and that the original prescription was supplied during the same calendar month.

It was reported that the cost of drugs and appliances for the first quarter of the medical year 1914 was about £60,000, whereas the total amount available for the year was only about £150,000. As it appeared that some practitioners prescribed unduly expensive or excessive drugs, a letter had been sent to every practitioner on the panel calling attention to the matter.

